# Advances in Thromboprophylaxis for High-Risk Pregnancies: A Comprehensive Review of Current Strategies and Emerging Approaches

**DOI:** 10.7759/cureus.67758

**Published:** 2024-08-25

**Authors:** Swati M Dahiphale, Deepika Dewani, Jayashree M Dahiphale, Manjusha Agrawal, Apoorva Dave, Sandhya Pajai, Garapati Jyotsna

**Affiliations:** 1 Obstetrics and Gynaecology, Jawaharlal Nehru Medical College, Datta Meghe Institute of Higher Education and Research, Wardha, IND; 2 Neurology, Fortis Hospital, Mumbai, IND

**Keywords:** low-molecular-weight heparins (lmwh), emerging therapies, pulmonary embolism (pe), deep vein thrombosis (dvt), pregnancy, thromboprophylaxis

## Abstract

Thrombosis during pregnancy poses a significant clinical challenge due to its potential for severe maternal and fetal complications. The incidence of thromboembolic events in pregnant women is heightened by pregnancy-associated hypercoagulability, venous stasis, and endothelial changes, all of which contribute to an elevated risk. Effective thromboprophylaxis is essential to mitigate these risks and improve outcomes for both mother and child. This review provides a comprehensive evaluation of current thromboprophylaxis strategies, including pharmacologic interventions such as low-molecular-weight heparins (LMWHs) and unfractionated heparin (UFH) and nonpharmacologic measures like compression stockings and lifestyle modifications. Additionally, the review explores emerging approaches, including personalized medicine strategies, novel anticoagulants, and technology-enabled monitoring solutions. By integrating current evidence with emerging trends, this review aims to offer insights into optimizing thromboprophylaxis in high-risk pregnancies, ultimately contributing to improved clinical outcomes and guiding future research directions in this critical area of maternal healthcare.

## Introduction and background

Thrombosis during pregnancy, which encompasses deep vein thrombosis (DVT) and pulmonary embolism (PE), poses a significant health risk, contributing to considerable maternal morbidity and mortality [1]. The incidence of thromboembolic events in pregnant women is approximately one in 1,000 pregnancies, highlighting the elevated risk compared to nonpregnant individuals. Pregnancy-induced changes such as hypercoagulability, venous stasis, and endothelial injury all contribute to this increased risk [2]. Specifically, the hypercoagulable state during pregnancy, driven by hormonal and hemodynamic changes, enhances the likelihood of clot formation. Furthermore, venous stasis due to the growing uterus compressing pelvic veins and reduced physical activity further exacerbates this risk. Identifying and managing these risks is crucial, as complications from thrombosis can range from relatively benign symptoms such as leg swelling and pain to severe outcomes like PE, which can lead to significant maternal and fetal complications [3].

Thromboprophylaxis plays a pivotal role in preventing thromboembolic complications in high-risk pregnancies. Effective prophylaxis strategies not only reduce the incidence of thromboembolic events but also help mitigate severe complications that can affect both maternal and fetal health [4]. For instance, appropriate use of thromboprophylaxis can decrease the likelihood of adverse outcomes such as preterm labor, intrauterine growth restriction, and placental abruption [5]. By enhancing maternal health and preventing the occurrence of venous thromboembolism (VTE), thromboprophylaxis contributes to better overall pregnancy outcomes, reduced hospitalizations, and improved neonatal health. In high-risk pregnant women, the benefits of implementing effective thromboprophylactic measures are profound, highlighting the importance of tailored and timely interventions to improve maternal and fetal well-being [5].

This review aims to provide a comprehensive overview of thromboprophylaxis strategies for high-risk pregnancies. It will begin by examining current practices, including established pharmacologic approaches such as low-molecular-weight heparins (LMWHs), unfractionated heparin (UFH), and nonpharmacologic measures like compression stockings and lifestyle modifications. The review will offer valuable insights into their role in preventing thrombosis by evaluating the efficacy, safety, and clinical application of these strategies. In addition, the review will explore emerging approaches in thromboprophylaxis, including advancements in personalized medicine, novel anticoagulants, and technology-enabled monitoring solutions. This exploration aims to provide a forward-looking perspective on how innovations in thromboprophylaxis could enhance care for high-risk pregnant women, ultimately leading to improved outcomes and optimized management practices.

## Review

Pathophysiology of pregnancy-associated thrombosis

Physiological Changes During Pregnancy

One of the most significant changes during pregnancy is the development of a hypercoagulable state, primarily resulting from alterations in the hemostatic system [6]. This hypercoagulability is marked by elevated levels of several coagulation factors, including fibrinogen (factor I), prothrombin (factor II), as well as factors VII, VIII, IX, X, and XII. Concurrently, there is a reduction in the levels of natural anticoagulants such as protein C and protein S, which are crucial for regulating coagulation [7]. Moreover, the activity of fibrinolytic pathways is diminished, leading to a decreased breakdown of clots. Enhanced platelet aggregation also occurs during pregnancy, further increasing the risk of thrombosis. These hemostatic changes are believed to have evolved to safeguard women from bleeding complications during childbirth but also render them more susceptible to thrombotic events [8]. In addition to these hemostatic changes, pregnancy induces specific alterations in the coagulation pathways. Notably, there is increased resistance to activated protein C, which normally functions to inhibit coagulation. Levels of von Willebrand factor and factor VIII are elevated, further facilitating clot formation. Protein S levels also decrease from the second trimester onward, contributing to the prothrombotic state. Collectively, these physiological changes create a favorable environment for thrombosis, especially when combined with other risk factors [9].

Risk Factors for Thrombosis

**Figure 1:**
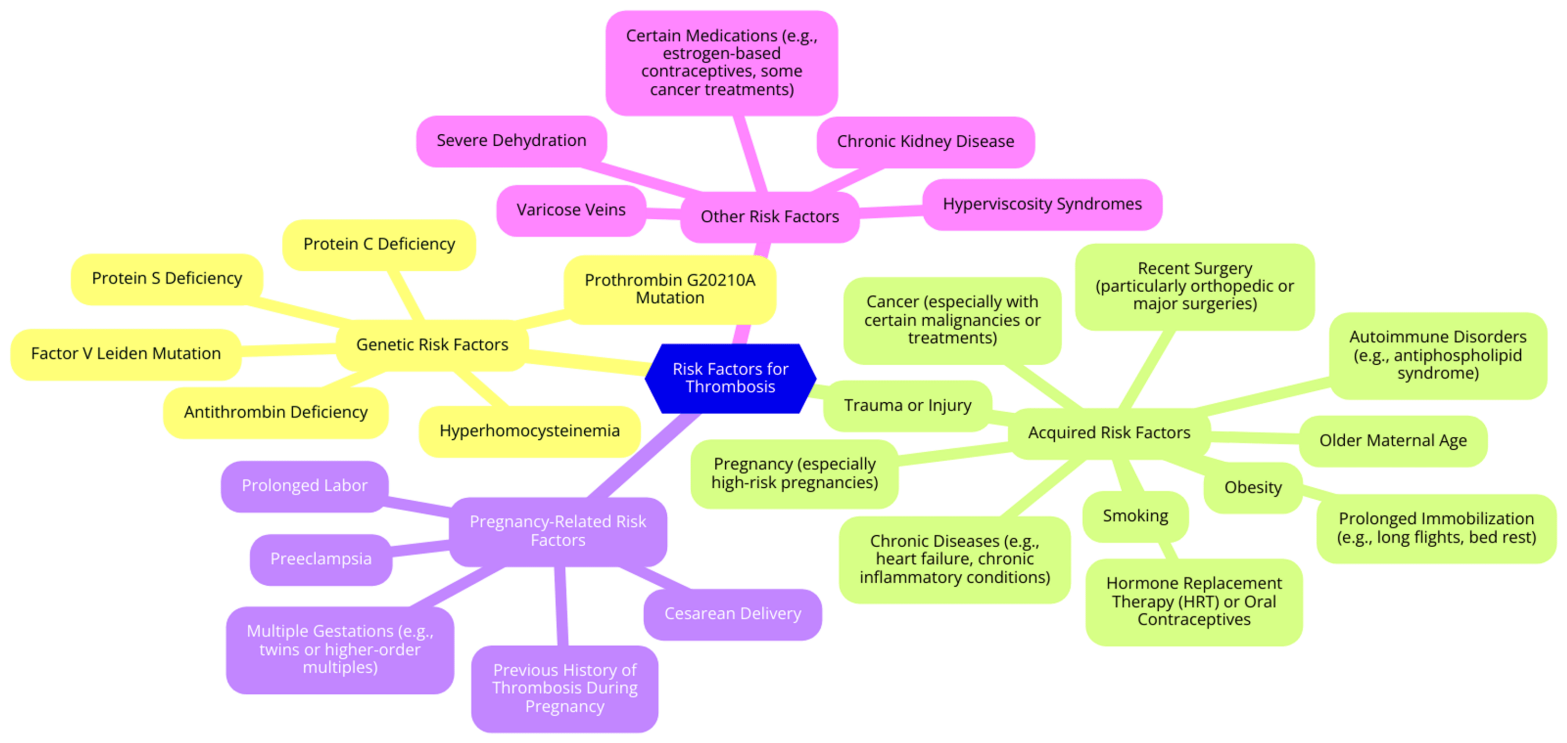
Risk factors for thrombosis Image credit: Dr. Swati Dahiphale

Several genetic and acquired risk factors can further elevate the risk of thrombosis associated with pregnancy. Genetic predispositions, such as the factor V Leiden mutation and the Prothrombin G20210A mutation, notably increase the likelihood of thromboembolic events. Additionally, deficiencies in natural anticoagulants like antithrombin, protein C, or protein S can further heighten the risk of thrombosis during pregnancy. These inherited conditions can interact with the physiological changes of pregnancy, amplifying the overall risk [10]. Acquired risk factors also play a significant role in the development of pregnancy-associated thrombosis. Obesity, for instance, is a major contributor due to its association with both mechanical factors (e.g., venous compression) and metabolic changes that promote a hypercoagulable state. Immobilization, whether from prolonged bed rest or extended travel, can exacerbate the risk of VTE. Additionally, smoking and advanced maternal age have been linked to an increased thrombotic risk during pregnancy [11]. Pregnancy-related risk factors are crucial for understanding the pathophysiology of thrombosis. Conditions such as multiple gestation pregnancies and preeclampsia significantly increase the risk of VTE. The postpartum period is particularly critical, as the risk of thrombosis can rise up to 20-fold during this time, especially following cesarean delivery. The interaction between pregnancy-induced hemostatic changes, coagulation pathway alterations, and various risk factors creates a highly thrombogenic environment, substantially increasing the risk of VTE during pregnancy and the postpartum period [12]. The risk factors for thrombosis are shown in Figure 1.

Current thromboprophylaxis strategies

Pharmacologic Interventions

LMWHs are extensively utilized for pharmacologic thromboprophylaxis in high-risk pregnancies. They primarily act by inhibiting factor Xa through the mediation of antithrombin III (AT-III). This inhibition prevents the conversion of prothrombin to thrombin, thereby obstructing the formation of fibrin from fibrinogen, a crucial step in clot formation. Common LMWH agents include enoxaparin (Lovenox) and dalteparin (Fragmin). For prophylaxis, the usual dosage of enoxaparin is 40 mg once daily or 30 mg twice daily, whereas treatment for VTE may require higher doses, such as 1 mg/kg every 12 hours or 1.5 mg/kg once daily, depending on the patient’s weight and renal function [13]. Regarding efficacy and safety, LMWHs have proven to be as effective as UFH in preventing and treating VTE in various populations, including pregnant women. The primary adverse effect associated with LMWHs is bleeding; however, they generally have a lower incidence of thrombocytopenia compared to UFH. Routine monitoring is usually unnecessary unless specific patient factors, such as renal impairment or obesity, are present [4]. UFH is another anticoagulant used for thromboprophylaxis, particularly in acute settings or hospitalized patients. It is indicated for the treatment of VTE and can be administered intravenously or subcutaneously, with the latter route commonly used for prophylaxis. A key consideration with UFH is the need for careful monitoring due to its variable pharmacokinetics. The activated partial thromboplastin time (aPTT) is typically used to guide dosing, with adjustments made based on aPTT results to maintain therapeutic ranges that ensure efficacy while minimizing bleeding risk [14]. Fondaparinux, a synthetic anticoagulant, selectively inhibits factor Xa and is FDA-approved for preventing DVT in surgical patients and for treating DVT and PE. It is also used off-label for patients with heparin-induced thrombocytopenia (HIT) due to its low risk of inducing this condition. Fondaparinux has a longer half-life and does not require routine monitoring, making it convenient for outpatient use. However, its use in pregnant women is generally limited due to insufficient safety data compared to LMWHs, which remain the preferred choice in this population [15]. Direct oral anticoagulants (DOACs), such as rivaroxaban and apixaban, have demonstrated effectiveness in nonpregnant populations, but their use during pregnancy is controversial. Current evidence regarding the safety of DOACs in pregnant women is limited, leading to recommendations against their use in this population. Potential risks of DOACs during pregnancy include teratogenic effects and bleeding complications. Consequently, LMWH remains the standard for thromboprophylaxis in high-risk pregnancies to ensure both maternal and fetal safety [16].

Nonpharmacologic Interventions

In addition to pharmacological approaches, nonpharmacologic interventions are crucial for reducing the risk of VTE in high-risk pregnant women. Two key strategies include the use of compression stockings and lifestyle modifications. Compression stockings are designed to apply graduated pressure to the legs, which enhances venous return and reduces venous stasis [17]. Studies have demonstrated their efficacy in lowering the risk of VTE during pregnancy. By promoting blood flow and preventing blood pooling in the lower extremities, compression stockings can significantly reduce the likelihood of clot formation [2]. Guidelines from organizations such as the American College of Obstetricians and Gynecologists (ACOG) advocate for the use of compression stockings, particularly for women with additional risk factors for VTE, such as obesity or prolonged immobility. Graduated compression stockings with a compression level of 20-30 mmHg are typically recommended, and they should be worn throughout the day, especially during prolonged periods of sitting or standing [18]. Lifestyle modifications are also essential components of a comprehensive thromboprophylaxis strategy. Maintaining a healthy weight during pregnancy is vital, as obesity is a significant risk factor for VTE. Excess weight can increase venous pressure and impair blood flow [19]. Women should follow a balanced diet rich in nutrients, manage caloric intake, and seek personalized weight management plans from healthcare providers. Regular monitoring of weight gain during pregnancy can help ensure it stays within recommended limits. Engaging in regular physical activity is another important aspect. Exercise enhances circulation, improves overall cardiovascular health, and reduces the risk of VTE [20]. It also helps maintain a healthy weight and alleviates some pregnancy-related discomfort. Pregnant women should aim for at least 150 minutes of moderate-intensity aerobic activity per week, as recommended by the World Health Organization (WHO). Activities such as walking, swimming, and prenatal yoga are beneficial. It is essential for women to consult their healthcare providers before starting any new exercise regimen, especially if they have pre-existing conditions or complications [21].

Emerging approaches and innovations

Personalized medicine is transforming thromboprophylaxis by leveraging an individual’s genetic profile to guide decisions on prevention, diagnosis, and treatment. In high-risk pregnancies, genetic testing can pinpoint patients with hereditary predispositions to VTE [22]. By understanding a patient’s genetic risks, healthcare providers can select the most suitable anticoagulants and determine optimal dosages. This shift from a "one size fits all" approach to a tailored treatment plan aims to enhance both the efficacy and safety of thromboprophylaxis [23]. Risk stratification models are also vital in identifying patients who need thromboprophylaxis. These models evaluate various risk factors, including genetic predispositions, family history of VTE, and other clinical indicators. Integrating genetic data into these models improves the accuracy of VTE risk assessments, enabling more targeted interventions and ensuring that high-risk patients receive appropriate preventive measures [24]. The development of novel anticoagulants represents another promising advance in thromboprophylaxis, offering potentially more effective and safer options for managing VTE risk in high-risk pregnancies. These new agents aim to improve upon existing therapies by reducing bleeding risks and providing more convenient dosing regimens. However, challenges remain, particularly the need for extensive clinical trials to establish the safety and efficacy of these new anticoagulants in pregnant populations [25]. Additionally, advancements in reversal agents for anticoagulants are crucial for managing bleeding complications. New reversal agents are being designed to rapidly counteract the effects of anticoagulants, enhancing patient safety. Improving the safety profiles and efficacy of these agents is especially important for high-risk populations, such as pregnant women, where the risks associated with anticoagulation therapy must be meticulously managed [26].

Technology-enabled monitoring is emerging as a valuable tool in managing thromboprophylaxis. Digital health solutions, including mobile applications and telemedicine, facilitate real-time communication between healthcare providers and patients. These technologies enhance adherence to treatment plans and allow for timely adjustments based on individual patient needs [27]. They also assist in tracking symptoms and potential side effects, improving overall management and ensuring patients receive necessary support throughout their treatment. Moreover, advances in biomarkers and novel diagnostic tools are enhancing the ability to identify high-risk pregnancies. New diagnostic methods provide critical insights into coagulation profiles and other relevant factors, enabling more precise risk assessments [28]. This information is crucial for making informed decisions about the necessity and intensity of thromboprophylaxis, ensuring that high-risk patients receive tailored care that meets their specific needs [28]. Table 1 summarizes emerging approaches and innovations in thromboprophylaxis for high-risk pregnancies.

**Table 1:** Emerging approaches and innovations in thromboprophylaxis for high-risk pregnancies

Category	Description	Examples/details	Potential benefits	Challenges/considerations
Personalized medicine	Tailoring prophylaxis based on individual risk factors and genetic profiles	-Genetic testing for thrombophilia; risk stratification models	-More targeted and effective prophylaxis; reduced risk of adverse events	-Accessibility and cost of genetic testing; variability in genetic risk assessment
Novel anticoagulants	Development of new anticoagulant agents with improved safety and efficacy profiles	-Direct oral anticoagulants (DOACs); new classes of anticoagulants in research	-Potential for safer and more effective options; reduced bleeding risk	-Limited data on safety during pregnancy; need for specific dosing guidelines
Reversal agents	New agents or improvements in existing agents to reverse anticoagulation effects	-New-generation reversal agents for DOACs; improved heparin reversal strategies	-Enhanced management of bleeding complications; more control over anticoagulation	-Availability and cost of new agents; integration into clinical practice
Technology-enabled monitoring	Use of digital tools and biomarkers for monitoring and managing thrombosis risk	-Mobile apps for tracking anticoagulant use; wearable devices for monitoring vitals; biomarkers for early detection of thrombosis	-Real-time monitoring and intervention; improved patient adherence and outcomes	-Privacy and data security concerns; implementation and integration into existing systems
Advances in imaging	Improved imaging techniques for better diagnosis and management of thrombosis	-Enhanced ultrasound technologies; magnetic resonance imaging (MRI) advancements	-More accurate detection of thromboembolic events; better assessment of treatment efficacy	-Cost of advanced imaging techniques; limited availability in some settings
Integrated care approaches	Comprehensive management strategies combining pharmacologic and nonpharmacologic methods	-Multidisciplinary care teams; combined use of medications and lifestyle interventions	-Holistic approach to care; improved overall outcomes and patient satisfaction	-Coordination among healthcare providers; potential for increased complexity in care

Challenges and controversies

Thromboprophylaxis in high-risk pregnancies involves several challenges and controversies that must be addressed to optimize patient outcomes. One primary challenge is balancing the benefits of anticoagulation with the risks of bleeding [29]. Anticoagulants, such as LMWH, are effective in significantly reducing the risk of VTE, which can lead to severe complications like PE, especially in high-risk populations. However, these medications also increase the risk of bleeding, which can be particularly concerning during pregnancy and childbirth. Healthcare providers must carefully evaluate these risks and benefits, engaging in detailed discussions with patients to ensure informed decision-making [29]. Another major challenge is the variability in thromboprophylaxis guidelines across different countries and organizations. Professional bodies, including the Royal College of Obstetricians and Gynaecologists (RCOG), the ACOG, and others, have developed their own guidelines based on differing interpretations of available evidence. This variability can lead to confusion among healthcare providers and patients regarding best practices for thromboprophylaxis. The lack of a unified approach may result in inconsistent patient care, where some women receive inadequate prophylaxis while others might be overtreated. Efforts are ongoing to harmonize these guidelines to ensure that all pregnant women receive optimal care based on the best available evidence [30]. Patient adherence to prescribed thromboprophylaxis regimens is crucial for preventing VTE but remains a challenge in clinical practice. Factors affecting adherence include a lack of understanding about the importance of the medication, concerns about side effects, complex dosing regimens, and issues related to forgetfulness or lifestyle [31]. To improve adherence, healthcare providers can implement several strategies: offering clear, accessible information about the risks of VTE and the benefits of thromboprophylaxis, simplifying dosing schedules and using user-friendly delivery methods, and conducting regular follow-up appointments to address concerns and reinforce the importance of adherence [32]. Table 2 summarizes the key challenges and controversies in thromboprophylaxis for high-risk pregnancies.

**Table 2:** Key challenges and controversies in thromboprophylaxis for high-risk pregnancies

Challenge/controversy	Description	Potential solutions/considerations
Risk vs. benefit analysis	Balancing the benefits of thromboprophylaxis against the potential risks of bleeding and other adverse effects	Use of individualized risk assessment tools; careful monitoring and dose adjustment
Variability in guidelines	Differences in thromboprophylaxis guidelines across countries and organizations, leading to inconsistent practices	Harmonization of guidelines through international collaboration and consensus
Patient adherence and education	Ensuring patients adhere to thromboprophylaxis regimens and understand the importance of medication and lifestyle changes	Enhanced patient education and support; use of digital tools for reminders and monitoring
Management of high-risk groups	Identifying and managing diverse high-risk groups such as those with multiple gestations, genetic predispositions, or obesity	Development of targeted protocols for specific risk groups; personalized treatment plans
Impact of emerging therapies	Incorporating new anticoagulants and monitoring technologies into existing protocols while evaluating their safety and efficacy	Conducting robust clinical trials; updating guidelines based on new evidence
Economic and resource constraints	Cost of thromboprophylaxis medications and monitoring and the availability of resources in different healthcare settings	Cost-effectiveness analyses; resource allocation strategies; use of cost-effective options where possible

Future directions and research opportunities

As the understanding of thromboprophylaxis in high-risk pregnancies advances, several key areas for future research and development become evident. These focus on enhancing clinical practices, improving patient outcomes, and integrating various strategies to effectively manage thromboembolic risks [33]. One critical area for future research is the need for longitudinal studies to assess the long-term effects of thromboprophylaxis on both maternal health and fetal development. Research should investigate how anticoagulant therapy during pregnancy impacts future cardiovascular health, reproductive outcomes, and the risk of VTE in subsequent pregnancies. Comprehensive data on maternal and fetal outcomes, including complications such as preterm birth, low birth weight, and developmental issues, will provide valuable insights into the safety and efficacy of thromboprophylaxis strategies [34]. The rapidly evolving research landscape necessitates the development of updated clinical guidelines that reflect the latest evidence on thromboprophylaxis. This includes integrating findings from recent studies on risk assessment models, individualized treatment approaches, and the efficacy of various anticoagulants. Updated guidelines should aim to standardize practices across healthcare settings to ensure that all high-risk pregnant women receive appropriate, evidence-based thromboprophylaxis. Such standardization can help reduce variability in care and improve outcomes [35]. Future research should also explore the potential benefits of combining pharmacologic interventions, such as LMWH, with nonpharmacologic strategies, including lifestyle modifications, physical activity, and compression therapy. Integrative approaches may enhance overall efficacy in preventing VTE and improving maternal health. Developing patient-centered care models that incorporate both pharmacologic and nonpharmacologic strategies can empower women to actively participate in their care. This may involve education on risk factors, lifestyle changes, and the importance of adherence to prescribed prophylaxis [36]. The future of thromboprophylaxis in high-risk pregnancies lies in a comprehensive approach that includes longitudinal studies, updated clinical guidelines, and integrative strategies. By addressing these areas, researchers and healthcare providers can work toward optimizing thromboprophylaxis protocols, ultimately improving maternal and fetal outcomes in this vulnerable population [33]. Table 3 outlines future directions and research opportunities in thromboprophylaxis for high-risk pregnancies.

**Table 3:** Future directions and research opportunities in thromboprophylaxis for high-risk pregnancies

Research area	Description	Potential impact
Longitudinal studies	Research examining the long-term outcomes of thromboprophylaxis in pregnant women and their children	Understanding long-term efficacy and safety, optimizing long-term care strategies for both mother and child
Development of new guidelines	Incorporation of recent evidence into updated clinical guidelines for thromboprophylaxis	Ensuring guidelines reflect the latest advancements, improving standardization of care
Genetic and personalized medicine	Use of genetic testing to tailor thromboprophylaxis to individual risk profiles	Enhanced precision in preventing thromboembolic events, reducing unnecessary treatment
Novel anticoagulants	Evaluation of new anticoagulant agents in pregnancy	Potential for safer, more effective options with fewer side effects compared to current medications
Reversal agents for anticoagulants	Development of effective reversal agents for novel anticoagulants used during pregnancy	Improving safety by providing options for managing bleeding complications associated with new therapies
Technology-enabled monitoring	Integration of digital health solutions for monitoring and managing thromboprophylaxis	Enhanced patient adherence, better monitoring of thromboembolic risk, and improved management
Biomarkers and diagnostic tools	Research into new biomarkers and diagnostic tools for identifying high-risk pregnancies more accurately	Improved risk stratification, enabling targeted thromboprophylaxis and better patient outcomes
Comparative effectiveness studies	Comparing the effectiveness and safety of different thromboprophylaxis strategies in diverse populations	Informing best practices and guiding individualized treatment plans based on comparative evidence
Integration of multidisciplinary approaches	Collaboration between obstetricians, hematologists, and other specialists in managing high-risk pregnancies	Comprehensive care strategies that address multiple aspects of thrombosis risk and prevention

## Conclusions

In conclusion, effective thromboprophylaxis in high-risk pregnancies is essential for preventing the serious complications associated with thrombosis, such as DVT and PE. The review highlights that while established strategies, including LMWHs and compression stockings, have demonstrated significant benefits in reducing thromboembolic events and improving maternal and fetal outcomes, there is a pressing need for ongoing advancements. Emerging approaches, such as personalized medicine and novel anticoagulants, offer promising avenues for optimizing prophylaxis and tailoring interventions to individual risk profiles. As research progresses and new technologies emerge, integrating these innovations into clinical practice will be crucial for enhancing the management of high-risk pregnancies. By continually refining our strategies and embracing new developments, we can improve the safety and health outcomes for both mothers and their babies, ensuring more effective prevention of pregnancy-related thrombosis.
